# NgAP2a Targets *KCS* Gene to Promote Lipid Accumulation in *Nannochloropsis gaditana*

**DOI:** 10.3390/ijms251910305

**Published:** 2024-09-25

**Authors:** Yihua Lin, Yanyan Li, Xiaobin Wu, Weinan Xu, Zhengying Zhang, Hongmei Zhu, Hantao Zhou

**Affiliations:** 1 State Key Laboratory of Marine Environmental Science, Xiamen University, Xiamen 361102, China; linyihua@stu.xmu.edu.cn (Y.L.); yanyanli2016@stu.xmu.edu.cn (Y.L.); wuxiaobin@stu.xmu.edu.cn (X.W.); xuweinan@stu.xmu.edu.cn (W.X.); zhangzhengying@stu.xmu.edu.cn (Z.Z.); 2 College of Ocean and Earth Sciences, Xiamen University, Xiamen 361102, China; flyzhu324@163.com; 3 State-Province Joint Engineering Laboratory of Marine Bioproducts and Technology, Xiamen University, Xiamen 361102, China

**Keywords:** *Nannochloropsis gaditana*, lipid, NgAP2a transcription factor, *KCS*

## Abstract

The commercialization of algal lipids and biofuels remains impractical due to the absence of lipogenic strains. As lipogenesis is regulated by a multitude of factors, the success in producing industrially suitable algal strains through conventional methods has been constrained. We present a new AP2 transcription factor, designated as NgAP2a, which, upon overexpression, leads to a significant increase in lipid storage in *Nannochloropsis gaditana* while maintaining the integrity of other physiological functions. These provide methodologies for enhancing petroleum output and optimizing the carbon fluxes associated with specific products. An integrated analysis of RNA sequencing (RNA-seq) and chromatin immunoprecipitation sequencing (ChIP-seq) data has elucidated that the NgAP2a-induced up-regulation of critical genes is implicated in lipogenesis. Specifically, NgAP2a has been demonstrated to directly bind to the M1 motif situated within the promoter region of the *KCS* gene, thereby promoting the transcriptional activation of genes pertinent to lipid metabolism. In summary, we elucidate a plausible pathway whereby NgAP2a serves as a direct modulator of the *KCS* gene (Naga_100083g23), thereby influencing the expression levels of genes and molecules associated with lipid biosynthesis.

## 1. Introduction

Microalgae are one of the primary producers on Earth, playing a significant role in the global carbon cycle [1]. By fixing carbon through microalgae, light energy and carbon dioxide can be converted into storage compounds such as proteins, lipids, polysaccharides, and pigments [2,3]. This process not only reduces carbon emissions but also provides various high-added-value raw materials for food, pharmaceuticals, energy, healthcare, and other fields [4,5,6,7,8]. *Nannochloropsis* is increasingly recognized as an excellent chassis organism for cell factories due to its rapid growth rate, ease of cultivation, and the maturity of its genetic manipulation tools [9,10]. It is progressively evolving into a patterned microalgae with potential for industrial oil production applications [11,12]. *Nannochloropsis* represents a promising light-driven cell factory in the realm of synthetic biology. The inherent ability to synthesize abundant lipids and valuable pigments makes *Nannochloropsis* an ideal candidate for the sustainable production of biofuels and high-value compounds [13]. However, in the industrial production of *Nannochloropsis*, high production costs remain a constraining factor. Therefore, cultivating algae strains with a high lipid content has become an important focus of current research. As a result, substantial resources have been allocated towards modifying microalgae by focusing on multiple pivotal metabolic nodes such as 1-deoxy-D-xylulose 5-phosphate synthase (DXS) [14], glucose-6-phosphate dehydrogenase (G6PD) [15], and diacylglycerol acyltransferase for lipid overproduction [16,17]. The availability of complete genome sequences and in silico identification of transcription factors (TFs) and their binding sites has opened up new opportunities for identifying potential metabolic targets that could enhance the industrial utility of certain microalgal strains [18]. AP2/ERF family transcription factors have been reported to be involved in regulating the synthesis of a variety of primary and secondary metabolites, which are important for plant growth and development [19,20,21]. Previous studies have shown that WRI1, a member of the AP2/ERF family of transcription factors, functions as an activator of genes involved in carbon metabolism and be required for accumulation of triacylglycerols (TAGs) in Arabidopsis seeds [22,23]. The WRI1 protein specifically interacts with a group of sequences located upstream of genes implicated in fatty acid biosynthesis, which collectively exhibit a conserved sequence motif characterized by the pattern CnTnG7[CG], with “n” denoting any nucleotide and the term “AW-box” being assigned to this particular sequence element [22]. Nevertheless, the literature contains a paucity of experimental studies elucidating the molecular characterization of transcription factors belonging to the AP2/ERF superfamily, as well as their specific roles and functional implications within the context of microalgae.

We identified a AP2 TF (designated NgAP2a) in *Nannonchloropsis gaditana* and investigated its function by overexpression and RNA interference (RNAi)-mediated silencing techniques in this study. Mechanistically, we elucidate the underlying molecular mechanisms through which the transcription factor NgAP2a directly interacts with the promoter of the *KCS* gene, subsequently enhancing the expression of genes associated with lipid metabolism. This process leads to an elevation in the levels of metabolic intermediates such as Glycerol-3-Phosphate (GAP), and Triacylglycerols (TAG). Additionally, it results in modifications to the physiological phenotypes, particularly those related to lipid content and overall sugar concentrations. Collectively, we describe a possible pathway in which NgAP2a acts as a direct regulator of the *KCS* gene, thereby affecting the expression levels of genes and molecules associated with lipid biosynthesis.

## 2. Results

### 2.1. Expression Vector Construction of NgAP2a

**Figure 1:**
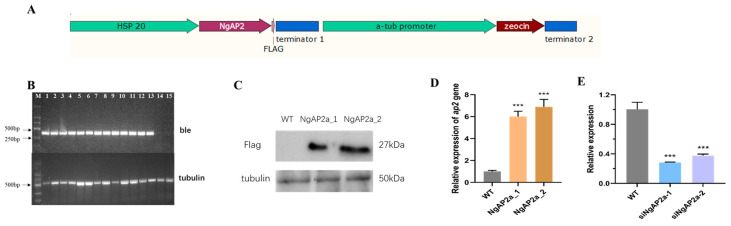
Identification of NgAP2a-engineered cells. (**A**) Schematic map of the NgAP2a overexpression cassette. (**B**) PCR identification of NgAP2a-engineered cells and WT. M indicates marker, lanes 1–5 are NgAP2a overexpression strains, lanes 6–13 are NgAP2a RNAi strains, and 14 and 15 are WT strains (negative control). (**C**) Western blot of NgAP2a-overexpressed cells and WT. (**D**) Transcriptional analysis of AP2 gene in NgAP2a-overexpressed strains. (**E**) Transcriptional analysis of AP2 gene in NgAP2a RNAi strains. Error bars represent mean values ± SD for three separate experiments. Significant difference is indicated at *p* < 0.001 (***) level.2.2. Overexpression of NgAP2a Altered Lipid and Carbohydrate Content without Affecting Photosynthetic Properties.

We overexpressed and silenced NgAP2a to characterize the role of NgAP2a in governing the expression of key metabolic nodes in *N. gaditana*. First, we cloned NgAP2a with two AP2 domains from the genome of *N. gaditana*, and NgAP2a was fused with a FLAG tag into the expression vector under the control of the Hsp20 promoter (Figure 1A). The recombinant expression vector was electroporated into *N. gaditana*. We silenced NgAP2a by using an RNA interference (RNAi) strategy. The construction of RNAi vectors involves the ligation of the full-length target gene sequence and a partial reverse sequence of the target gene to form a hairpin structure, with unmatched segments of the full-length sequence serving as loop structures (Supplemental Figure S1).

Resistant colonies were inoculated into F/2 medium, and the culture was sub-cultured regularly at 7-day intervals for over 6 months. The transformants were also maintained on a medium containing zeocin (2 μg/mL), and their growth was observed to verify the stability of transformants. The wild-type (WT) strain could not survive in the media with antibiotics, while transformants exhibited robust growth (Supplemental Figure S2).

Subsequently, putative algal transformants were initially screened by genomic polymerase chain reaction (PCR). Notably, the preliminary screening of genetically engineered algal strains expressing NgAP2a revealed successful amplification of the *ble* gene (375bp), while the WT strain failed to do so (Figure 1B). The successful amplification of the tubulin gene across all tested algal strains suggests the integrity of the algal DNA template (Figure 1B). The preliminary screened transformants were further evaluated by means of quantitative PCR and Western blotting. A Western blot analysis indicated the presence of a cross-reacting band with a molecular weight of approximately 27 kDa in the transformed cell samples (Figure 1C). This observation aligns with the predicted size of NgAP2a, suggesting successful expression of the gene within overexpressing cell lines.

A 6.01- and 6.89-fold increase in the relative transcript levels of NgAP2a was observed in the engineered cell lines NgAP2a_1 and NgAP2a_2, respectively, compared to the control, as determined by a quantitative PCR (qPCR) analysis (Figure 1D). However, the relative transcript level of silenced strains siNgAP2a_1 and siNgAP2a_2 was decreased by 0.28- and 0.37-fold compared to WT (Figure 1E). The absence of such bands in the WT suggests that the observed band specifically corresponds to the recombinant NgAP2a protein rather than endogenous proteins. The consistency between the experimental data and the theoretical expectations provides strong evidence for the successful manipulation of NgAP2a expression in the engineered cell lines.

**Figure 2:**
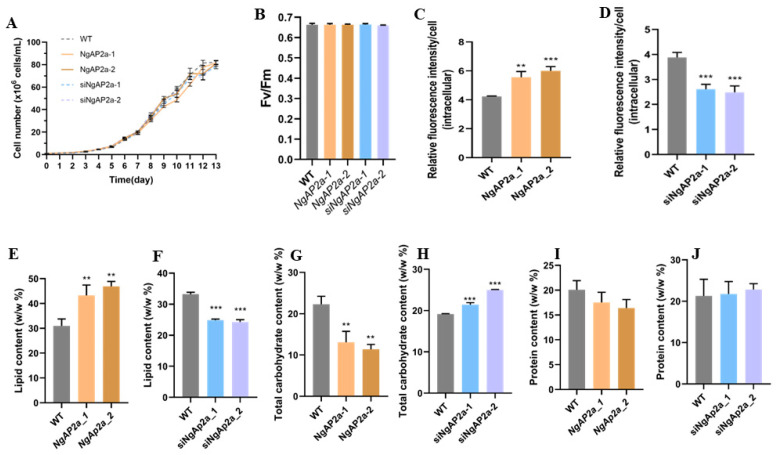
Physiological and biochemical analysis of NgAP2a-engineered cells. (**A**) Growth curve analysis. (**B**) Maximum quantum yield of photosystem II (PSII) as measured by Fv/Fm. Relative fluorescence intensity of intracellular lipids as determined by Nile red fluorescence analysis per cell among (**C**) NgAP2a-overexpressed cells and WT, and (**D**) RNAi cells and WT. Intracellular lipid content (percentage of total cell dry weight) of (**E**) overexpressed strains and (**F**) RNAi strains. Total carbohydrate content, as determined by the phenol-sulfuric acid method of (**G**) overexpressed strains and (**H**) RNAi strains. Total protein content, as measured by bicinchoninic acid (BCA) assay of (**I**) overexpressed strains and (**J**) RNAi strains. Error bars represent mean values ± SD for three separate experiments. Significant difference is indicated at *p* < 0.01 (**) or *p* < 0.001 (***) level.

As shown in Figure 2, there was no change in photosynthesis, and growth was observed between the overexpressing and WT strains. This was determined by the maximum quantum yield of photosystem II (PSII) (Fv/Fm), a sensitive method for assessing the photosynthetic efficiency of PSII, which revealed that no significant change in Fv/Fm was observed between the WT and overexpressing cells (Figure 2B). Furthermore, we detected the relative lipid content by using Nile red fluorometric analysis and found that the specific increase in lipid content observed in the two strains of algae overexpressing NgAP2a, when compared to WT, amounted to an enhancement of 31.19% and 41.70%, respectively (Figure 2C). However, lipid levels in RNAi lines were significantly lower than in the WT (Figure 2D). To further confirm and evaluate the function of NgAP2a-overexpressing strains in the lipid metabolism pathway, we analyzed the percentage of lipids, proteins, and carbohydrates in algal cells at the early stage of the platform in terms of the dry weight of the cells. NgAP2a overexpression increased the intracellular lipid percentage by about 39.84–51.71% compared to WT, and the magnitude of the increase was less consistent across strains (Figure 2E). Meanwhile, the carbohydrate percentage was significantly reduced by about 33.69–39.96% relative to WT, while there was no difference in protein content (Figure 2G,I), whereas RNAi strains showed the opposite phenotype (Figure 2F,H,J).

### 2.2. NgAP2a Up-Regulated the Expression of Lipid-Related Genes

**Figure 3:**
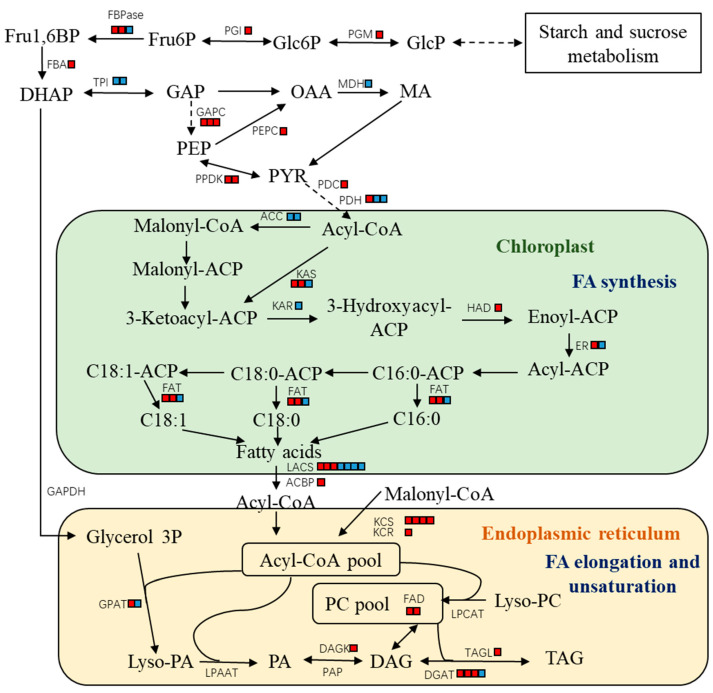
Changes in genes related to glycolysis and lipid biosynthesis in NgAP2a-overexpressed strain. Note: The red and blue boxes represent the significantly up-regulated or down-regulated expression of each enzyme at each stage respectively (*n* = 3, *p* < 0.05), and multiple boxes represent that the enzyme has multiple significantly differentially expressed homologous genes, Solid lines represent one-step reactions and dashed lines represent multi-step reactions. The transcript IDs of the relevant genes were as follows: PGM, Naga_100150g10; PGI, Naga_100003g157; FBPase, Naga_100011g32, Naga_102887g1 and Naga_101891g1; FBA, Naga_100154g7; TPI, Naga_100001g84 and Naga_103287g1; MDH, Naga_100855g2; GAPC, Naga_100081g16, Naga_100257g1, Naga_100081g17; PEPC, Naga_100016g66; PPDK, Naga_100043g42, Naga_100134g1; PDC, Naga_100009g44; PDH, Naga_100130g6, Naga_100065g5, Naga_100006g108; ACC, Naga_101049g3 and Naga_100061g16; KAS, Naga_101498g1, Naga_100086g24 and Naga_100004g8; KAR, Naga_100212g8; HAD, Naga_100113g7; ER, Naga_100031g35 and Naga_101053g1; FAT, Naga_100017g31, Naga_100782g1 and Naga_100884g2; LACS, Naga_100012g66, Naga_100495g2, Naga_100035g43, Naga_100022g68, Naga_101051g1, Naga_100021g41 and Naga_100713g1; ACBP, Naga_100042g16; KCS, Naga_100083g23, Naga_100004g102, Naga_100003g8 and Naga_100608g3; KCR, Naga_100004g168; FAD, Naga_100115g11 and Naga_100545g1; GPAT, Naga_100002g46 and Naga_100154g3; DAGK, Naga_100002g79; DGAT, Naga_100018g41, Naga_100028g44, Naga_100010g31 and Naga_100251g8; TAGL, Naga_100057g25.

As shown in Figure 3, the overexpression of NgAP2a caused a trend towards the up-regulated expression of most genes of the glycolysis, fatty acid synthesis, fatty acid elongation, and unsaturation pathways, and a trend towards the differential expression of multiple transcripts of some of the genes, similarly to the results described in the article by Südfeld et al. [24]. As the final step in TAG biosynthesis, *DGAT* has four homologous genes showing differential expression, three of which are up-regulated. However, the two significantly differentially expressed acetyl-CoA carboxylase (*ACC*) genes in this study were down-regulated after NgAP2a overexpression, and no significantly differentially expressed pyruvate kinase (*PK*) genes were found, revealing that NgAP2a differs from the *Arabidopsis* regulatory mechanism. The target genes to which NoAP2 may bind have been postulated by bioinformatics to be ketoacyl ACP synthase (KAS) (type I), phosphatidic acid phosphatase (PAP), and long-chain acetyl coenzyme A synthetase (LACS) [18], and *LACS* genes were also found among the differentially expressed genes in this study, with three of seven transcripts up-regulated, and the *KAS* type I encoded by Naga_100086g24 was up-regulated (*p* = 1.40 × 10^−9^) and all four genes encoding *KCS* were up-regulated. Therefore, we hypothesize that these genes may be directly regulated by NgAP2a. Kang et al. (2017) transformed *Arabidopsis* AtWRI1 into *Nannochloropsis salina*, and the transformed AtWRI1 up-regulated *PPDK* (pyruvate phosphate dikinase), *LPL* (lysophospholipase), *LPGAT1* (lysophosphatidylglycerol acyltransferase), and *PDH* gene expression and down-regulated *TAGL* and *DAGK* gene expression, resulting in increased lipid content [25]. Among these promoter-containing AW-box genes, only *DGAT* (Naga_100028g44), and *PPDK* (Naga_100043g42) showed a consistent trend in NgAP2a-overexpressing strains in this study, which implies that the motifs bound by NgAP2a may be more stringent than the AW-box or completely different.

In addition, we found that the four homologs of the *KCS* gene differentially expressed in the fatty acid elongation and desaturation pathways (containing Naga_100083g23, Naga_100004g102, Naga_100003g8, and Naga_100608g3), which encodes the first step of fatty acid elongation and is annotated as a ketoacyl-CoA synthase and fatty acid elongation enzyme, were all up-regulated. Phosphatidylglycerol (PG) was up-regulated in the metabolome of the NgAP2a overexpressed strains, suggesting that NgAP2a transformation reserves sufficient precursors via the Kennedy pathway to contribute to TAG biosynthesis.

### 2.3. Chip Analysis Demonstrated the Role of NgAP2a in Governing the Expression of Key Genes in Lipid Metabolism

Subsequently, we aim to elucidate the NgAP2a regulatory mechanism of excess lipid production in overexpressing strains by chromatin immunoprecipitation (ChIP). The ChIP analysis revealed that NgAP2a regulates the expression of long-chain fatty acid synthase (LCFA), which is required for extension in fatty acid synthesis and involved in the lipid synthesis pathway. The ChIP-Seq showed some genes of glycolysis and fatty acid related pathways can be bound on the promoter region by NgAP2a, these genes mainly including LACS, GPAT, DGAT, and TAGL (Table S1). Combining the results of a transcriptome analysis, we found that the genes encoding LACS (Naga_100083g23, Naga_100004g102, and Naga_100608g3), DGAT (Naga_100010g31 and Naga_100028g44) and the PGM (Naga_100150g10) and PDH (Naga_100065g5) genes involved in glycolysis are present in both sets of omics data, suggesting that these genes may be direct target genes for NgAP2a regulation.

### 2.4. Identification of DNA Motifs for NgAP2a Binding

**Figure 4:**
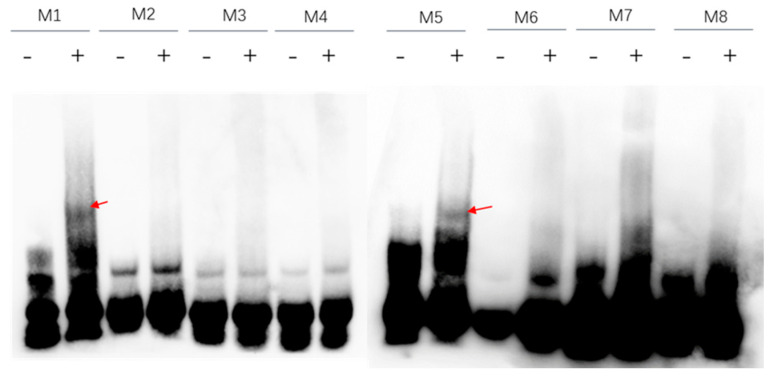
The combination detection of potential motifs and PET28-NgAP2a. Note: + and − indicate the presence or absence of purified PET28-NgAP2a protein, respectively, and the red arrow indicates the binding band between the probe and the protein.

We employed the computational tool known as Homer to conduct both pattern discovery and enrichment analyses of the identified peak regions across various samples. The threshold for peak count in the enrichment analysis was set at a minimum of 50 by default. Subsequently, based on the statistical significance of the enriched peaks, we tentatively shortlisted eight candidate motifs for further examination (Table 1). We synthesized probes from the eight potential motifs, marked them with biotin at their 3′ end, and through renaturation allowed the probes to form double-stranded structures. These probes were then co-incubated with purified proteins. As a negative control, we used free probes without added protein to determine whether they could bind to NgAP2a protein. As indicated in Figure 4, both the M1 and M5 motifs are able to bind to the purified PET28-NgAP2a protein (at the position marked by red arrows), whereas no bands appear at this location when only free probes are added (to the left of the red arrow lane). Other sequences cannot bind to the purified PET28-NgAP2a protein.

**Figure 5:**
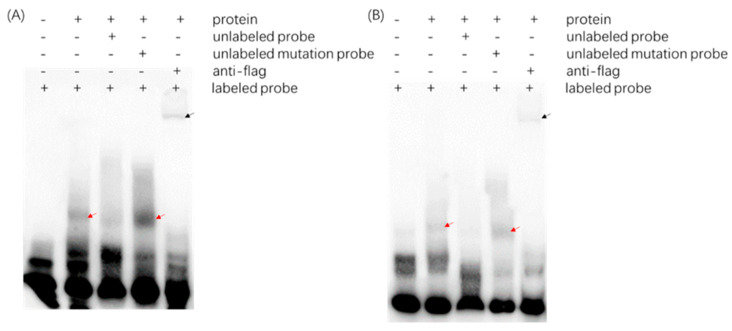
EMSA Analysis of PET28-NgAP2a protein binding to probes. (**A**) Co-incubation with purified PET28-NgAP2a protein using M1 motif; (**B**) co-incubation with purified PET28-NgAP2a protein using M5 motif. Note: Unlabeled probes use 100× specific probes, while unlabeled mutant probes also use 100× equivalent probes, but only the probe sequence is changed. Red arrows are bound bands and black arrows are supermigrated bands. The symbol “+” signifies that the substance on the right was included in the reaction solution for this particular lane, whereas the symbol “-” indicates that it was not added to the solution.

To investigate whether the M1 and M5 motifs truly bind to NgAP2a, we utilized specific cold competition probes for competitive binding assays. As shown in Figure 5, the incorporation of an excess (100-fold) of specific unlabeled cold probes resulted in the displacement of the binding band (lanes 3 in Figure 5A,B), whereas adding an equal amount (100-fold) of mutant probes failed to compete away the binding band (lanes 4 in Figure 5A,B). These findings collectively suggest that NgAP2a can bind to both M1 and M5 motifs, with the mutated base being essential for this interaction. Upon comparing the sequences of the two, we found that GCAA is a shared nucleotide sequence in both, which may serve as a critical core element for binding. After careful alignment, it was discovered that the M3 sequence also contains GCAA, yet this sequence is unable to bind with NgAP2a, suggesting that the nucleotides flanking GCAA are also of significant importance.

### 2.5. Target Gene Analysis for NgAP2a Binding

We further conducted a genome-wide search for genes with promoters containing the M1 or M5 motif, and the results indicated that the M1 motif is present in the promoter regions of two genes, Naga_100162g4 and Naga_100083g23. Both of these genes encode long-chain fatty acid synthases (KAS III enzymes), and the differential expression of the Naga_100083g23 gene was observed in the aforementioned transcriptome data. This finding further suggests that NgAP2a can directly regulate fatty acyl coenzyme A synthetase, thereby participating in the process of fatty acid synthesis and extension. The complete M5 motif is exclusively found in Naga_101276g1, a gene that encodes ribosomal protein I30, indicating the involvement of NgAP2a in the process of ribosomal protein synthesis.

**Figure 6:**
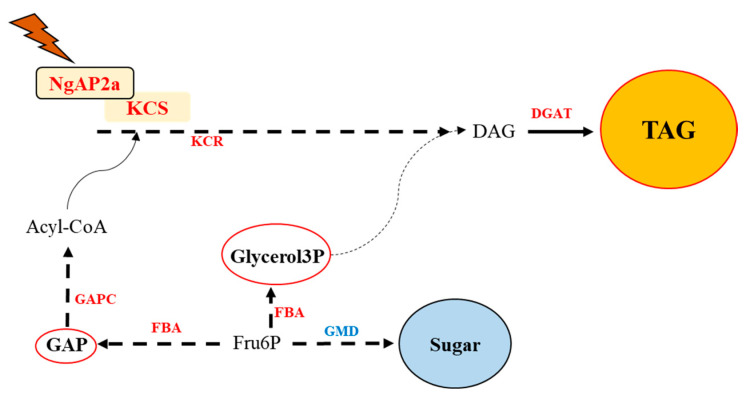
Schematic representation of the role of NgAP2a in *N. gaditana*. Note: Orange shading indicates substances significantly up-regulated in the physiological phenotype, blue shading indicates substances down-regulated, red font indicates genes significantly up-regulated in transcript levels, and blue font indicates genes significantly down-regulated in transcript levels. Red circles indicate that the substance is up-regulated in the metabolome. Solid lines indicate one-step reactions, and dashed lines indicate multi-step reactions.

Therefore, based on the aforementioned results, we describe NgAP2a as a transcription factor that directly binds to the TCTGCAAAGCYC motif in the promoter region of the KCS gene, leading to up-regulated expression of the KCS gene in the pathway for long-chain fatty acid synthesis and elongation. This up-regulation is accompanied by an increased expression of the ketoacyl-CoA reductase (KCR), fructose bisphosphate aldolase (FBA), and diacylglycerol acyltransferase (DGAT) genes, culminating in an increase in intracellular lipid, which aligns with the metabolomic data showing elevated levels of glyceraldehyde 3 phosphate (GAP), Glycerol-3-Phosphate (Glycerol3P), and triacylglycerol (TAG) (Figure 6). Furthermore, the overexpression of NgAP2a resulted in the down-regulation of GDP-mannose 4,6-dehydratase (GMD) gene expression, significantly reducing the total sugar content in the transgenic algal strain.

## 3. Discussion

These results indicated that NgAP2a overexpression led to a significant increase in intracellular lipid content at the expense of carbohydrate reduction, whereas knockdown led to a decrease in lipid content. However, a previous study showed that random insertion and disruption of the *AP2* gene in the *N. oceanic* strain IMET-1 led to an enhancement in photosynthetic efficiency and a substantial 40% rise in the content of neutral lipid [24]. This may be due to differences in algal species and culture conditions, as the culture conditions and media used in Südfeld et al. (2021) differed significantly from those used in this study. Phenotypic differences caused by the same gene in different species have also been reported in plants. For example, GmWRI1a expression in soybean resulted in an increase in oil content [26], whereas the overexpression of AtWRI1 into soybean resulted in little change in seed lipid content [27]. At the same time, the phenotype of complete loss of WRI1 (Atwri1RNAi) in *Arabidopsis* was similar to that observed in overexpressing lines co-expressing AtWRI1 and AtDGAT1 in soybean, which both showed similar physiological and biochemical responses, such as crumpled seed phenotypes, higher starch accumulation, higher glucose and fructose, and defects in the conversion of sugars to fatty acid (FA) [19,28]. Thus, perturbing lipid homeostasis in both directions leads to similar phenotypes. Liu et al. (2019) demonstrated that *Arabidopsis* WRI1 is regulating genes encoding FA synthesis and BADC (biotin-attachment domain-containing BIOTIN ATTACHMANT DOMAIN-CONTAINING) proteins, which are conditional inhibitors of FA (the proteins inhibit FA synthesis by long-term irreversible inhibition of the ACC enzyme in the presence of FA overdosage), revealing a coordinated mechanism to achieve lipid homeostasis [29]. WRI1, one of the AP2 family members, which encompasses transcription factors native to *Arabidopsis*, has been recorded in the literature to exhibit binding affinity towards the promoter region of BCCP2. Notably, BCCP2 serves as a crucial subunit within the intricate assembly of the ACC complex, a key metabolic enzyme [23]. And ACCase subunits are differentially regulated by WRI1 [30]. In addition, different members of the same TF family in *Nannochloropsis* have functional differences; e.g., NobZIP1 overexpression allows lipid accumulation [31], whereas NobZIP77 is thought to be a negative regulator of TAG synthesis [11]. These findings suggest the functional diversity of AP2/ERF family members, highlighting their complexity, and reveal that there exist functional disparities among different species. This underscores the varied roles and intricate nature of AP2/ERF proteins across species, demonstrating their evolutionary adaptability and specialization.

The strain engineered for the overexpression of NgAP2a was found, within the context of this investigation, to manifest a decreased carbohydrate content alongside an augmented lipid content, relative to WT, with no discernible impact on the inherent photosynthetic characteristics. Ohto et al. (2005) showed that *Arabidopsis* ap2 mutations cause changes in the ratio of hexose to sucrose during seed development, opening the possibility that AP2 may control seed mass through its effects on sugar metabolism [32]. In addition, the protein encoded by the *ANT1* (AINTEGUMENTA-LIKE1) gene in maize, which is an AP2 transcription factor, binds the promoters of GNC and AN3, which are key regulators of chloroplast development and plant growth [33]. It may be the potential link between the AP2 transcription factor and photosynthesis.

We showed that the overexpression of NgAP2a caused a trend towards the up-regulated expression of most genes of the glycolysis, fatty acid synthesis, fatty acid elongation, and unsaturation pathways, and a trend towards the differential expression of multiple transcripts of some of the genes, similarly to the results described in the article by Südfeld et al. [24]. Three of four *DGAT* homologous genes were up-regulated in this study; however, a previous report suggested that the *Arabidopsis* AP2 subfamily member WRI1 interacts directly with pyruvate kinase (PK), acetyl coenzyme A carboxylase (ACC), and ketoacyl ACP synthase (KAS) via the AW box, and that the expression of these genes is down-regulated in the WRI1 knockout mutant strain [22]. Van Erp et al. (2014) reported that in *Arabidopsis thaliana*, the enhancement of fatty acid content was more pronounced through the silencing of the triacylglycerol lipase *SDP1* gene and the overexpression of *WRI1* and *DGAT1*, compared to any single overexpression of *WRI1* or *DGAT1*, or the silencing of the *SDP1* lipase gene alone [34]. Thus, the WRI1 transcription factor does not act directly on a single gene to regulate plant oil content, but is involved in regulation through a complex network of simultaneous interactions with multiple metabolism-related genes [35]. Similarly, based on the above analyses, we suggest that the regulation of lipid content by NgAP2a is a complex process that does not rely solely on the regulation of transcription of individual genes, but may also involve a variety of post-transcriptional regulation, such as methylation modification, miRNA regulation, and so on.

*Nannochloropsis*, being ancient heterokont protists, evolved through secondary endosymbiosis from red algal plastids. The heterokonts (Stramenopiles), Alveolata, and Rhizaria are frequently grouped together as the SAR supergroup. Studies have indicated that the apicoplasts in members of the supergroup Alveolata, specifically within the phylum Apicomplexa, are derived from red algae and have lost their photosynthetic capabilities during evolution [36]. The apicomplexan Apetala2 (ApiAP2) have evolved into transcription factors that are capable of binding to the nucleotide sequence TGCATGCA [37]. Oberstaller et al. (2014) demonstrated that the ApiAP2 protein specifically binds to the DNA sequences TGCAT, CACACA, and the G-box (G(T/C)GGGG), thereby regulating the expression of target genes [38]. Despite significant differences between the NgAP2a and ApiAP2 transcription factors, the M1 sequence directly bound by NgAP2a in this study is TCTGCAAAGC (T/C), which shares four base pairs with the TGCAT sequence. Additionally, the reverse complement of the M5 sequence ATACCGCAAGTA contains four bases that are consistent with the G-box, namely GCGG. Hu et al. (2014) predicted that the DNA motif potentially bound by NoAP2 (NO06G03670) is rich in C/G bases, such as CGCGCCA (A/T), TCCGCCC (A/C), and GCC(G/C)ATCC [18]. Therefore, the consensus sequences recognized by AP2 transcription factors exhibit a degree of conservation across diverse organisms ranging from protozoa and cyanobacteria to higher plants, allowing them to directly bind to G-box motifs [39].

## 4. Materials and Method

### 4.1. Microalgae Culture Conditions

*N. gaditana* CCMP 526 was obtained from National Center for Marine Algae and Microbiota, USA. Microalgae were cultured in F/2 liquid medium at 22 °C in an artificial climate incubator with 50 μmol photons m^−2^ s^−1^ under a photoperiod of 12/12 h light/dark cycle, while being supplied directly with air containing 1.5% CO_2_.

### 4.2. Construction of NgAP2a Expression Vector

The NgAP2a (Genbank: EKU22783) was amplified from cDNA of *N. gaditana* with primers NgAP2a-F1 and NgAP2a-R1. Flag tag was designed in the primers and fused at the C terminal of the target genes for the detection of protein expression. The amplified product of NgAP2a was cloned into HSP expression vectors with Hsp20 promoter by using the ClonExpress II One Step Cloning Kit (Vazyme, Nanjing, China) to obtain recombinant overexpression vectors hsp-NgAP2a. The RNAi vector was engineered through the connection of the complete sequence of the target gene (long fragment) and the antisense segment of a portion of the target gene (short fragment), ensuring their complementarity to establish a hairpin structure. In the creation of these vectors, the initial step involves incorporating *Bam*HI and *Eco*RI endonucleases into their respective primer sequences. Following amplification, long and short fragments with *Bam*HI and *Eco*RI enzymes located at the 5’ and 3’ ends are obtained. These fragments are then inserted into the hsp-tub-zeo expression vector. Subsequently, the two fragments are fused together through double digestion using T4 ligase, allowing the 3’ ends of both the long and short fragments, which are both *Eco*RI sticky ends, to connect with the long fragment. This fusion results in the short fragment’s sequence becoming complementary to the 5’ end of the long fragment in reverse, forming a hairpin structure, with the unpaired sequence acting as a loop, thereby achieving a gene silencing effect. The vector contains a gene coding for resistance to zeocin (*Shble*), which expressed under the control of the tubulin promoter and terminator, allowing selection of transformed cells. The constructed overexpressed recombinant vectors and RNAi vectors were electroporated into microalgae using a Gene Pulser Xcell electroporation system (Bio-Rad, Hercules, CA, USA) as Li et al. described [40]. Primer sequences used in this study are listed in Table S2.

### 4.3. Molecular Identification of Transformants

The integration of the transgene into the host genome was confirmed by genomic PCR. An amount of 2 μL of *Nannochloropsis* cells was used as a template for genomic PCR. The *Shble* gene was detected by ble-F and ble-R primers, and the *tubulin* gene was detected by tub-F1 and tub-R1 primers to evaluate the DNA consistency (Supplemental Table S2). The PCR was amplified by 2×Accurate Taq master mix (Accurate Biotechnology Co., Ltd., Changsha, China) under the following conditions: 98 °C for 8 min, 35 cycles of 98 °C for 30 s, 60 °C for 40 s, 72 °C for 1 min, and then 72 °C for 10 min. The PCR products were assessed by agarose gel electrophoresis, and the sizes of the *Shble* and *tubulin* were 357 bp and 500 bp, respectively.

Total RNA was extracted from overexpressing strains, silencing cells, and WT by using the Polysaccharide/polyphenol Plant Total RNA Mini Kit (GeneBetter^®^, Beijing, China) according to the manufacturer’s instructions. First-strand complementary DNA was synthesized using an Evo M-MLV RT Kit with gDNA Clean for qPCR (Accurate Biotechnology Co., Ltd., Changsha, China). The efficacy of overexpression and silencing of the target genes in the microalgae cells were assessed by real-time qPCR using a SYBR^®^ Green Premix Pro Taq HS qPCR Kit (Accurate Biotechnology Co., Ltd., Changsha, China) on QuantStudio 6 Flex (Applied Biosystems, Inc., Carlsbad, USA) according to the manufacturer’s instruction. The transcripts of NgAP2a were determined by the primers qNgAP2a-F1 and qNgAP2a-R1. *Actin* gene was used as the internal control and the qPCR primer sequences are listed in Table S2. The mRNA levels were quantified using the double delta Ct method [41].

Western blotting was conducted using anti-Flag antibody (1:1000; Sigma-Aldrich, Darmstadt, Germany) to examine the expression of target proteins in the overexpressing cells since Flag-tag at the C-terminus of NgAP2a existed in the HSP expression vector. An aliquot of 20 μg protein from each sample was resolved by 12% SDS-PAGE, and then the gel was electro-transferred to a PVDF membrane for standard Western blot analysis. The anti-tubulin antibody was used as a reference (1:1000; Abcam, Cambridge, UK) and horseradish peroxidase-conjugated goat anti-mouse antibody (LABLEAD Inc., Beijing, China) at a dilution of 1:2000 was used as a secondary antibody.

### 4.4. Analysis of Growth and Photosynthetic Efficiency

After being cultivated in fresh and non-antibiotic medium several times, the engineered and WT cells were harvested and resuspended in fresh F/2 medium without zeocin. The initial algal cell concentration was made up to 1 × 10^6^ cells/mL. The growth curve was determined by counting cells by flow cytometry CytoFLEX S (Beckman, CA, USA) every day. Both the transformants and WT cells were incubated in dark for at least 20 min, and hence, the maximum photochemical efficiency of PSII (Fv/Fm) was measured by a Multi-excitation wavelength modulated chlorophyll fluorometer (Multi-Color-PAM, Walz, Nuremberg, Germany).

### 4.5. Analysis of Neutral Lipids and Fatty Acid Profile

An aliquot of cell cultures in the stationary phase were treated with 5%DMSO and 1 μg/mL Nile red (working concentration), and then mixed by rapid inversion and incubated at room temperature for at least 40 min in dark to promote the Nile red permeability. The fluorescence intensity of being stained cell cultures were measured by flow cytometry (CytoFLEX S, Beckman, CA, USA) with excitation and emission wavelengths of 488 nm and 585 nm, respectively, which could provide quantitative comparison of neutral lipid contents between transformants and WT. The total lipid content and fatty acid composition were determined by gravimetric analysis and gas chromatography-mass spectrometry (GC-MS), respectively, as previously described [42].

### 4.6. Measurement of Carbohydrate and Protein Content

Ten mg freeze-dried microalgae samples were resuspended in 0.5 mL of glacial acetic acid. The content was mixed thoroughly, followed by incubation in water bath at 85 °C for 20 min. Then, 10 mL of acetone was added to each tube, mixed well, centrifuged at 1000× *g* for 10 min, and carefully discarded the supernatant. Next, 2.5 mL of trifluoroacetic acid (4 M) was added to each sample tube and mixed by shaking. After capping the tubes, the content was hydrolyzed in a boiling water bath for 4 h. After the end, the samples were centrifuged at 12,000× *g* for 5 min and then the supernatant and different concentrations of glucose (used for standard curve) were mixed by the phenol-sulfuric acid solution (15 mL H_2_SO_4_: 7.5 mL H_2_O: 0.15 g phenol) with incubation at 100 °C for 20 min. As phenol reacted with carbohydrates, it turned an orange color and could be detectable at 490 nm. The total protein of the samples in the stationary phase was extracted by 1 M NaOH at 80 °C for 10 min with three times. The protein concentration was determined by a Bradford Protein Assay Kit (Solarbio, Beijing, China). All data were analyzed with one-way ANOVA (GraphPad Prism 8). Three experimental repeats were conducted.

### 4.7. mRNA-Seq and Metabolomics

For the transcriptomic analysis, microalgal cultures of WT and overexpressed strains were inoculated in log phase with 5.0 × 10^8^ cells per sample and 3 biological replicates per stain. Transcriptome sequencing was commissioned to Novozymes Technology Co. (Tianjin, China). The WT and overexpressing algal strains, namely NgAP2a_1, were cultivated under two distinct growth phases: the exponential phase commencing at day 4 post-inoculation (labeled as WT-log and AOE-log thereafter) and the stationary phase reached at day 12 post-inoculation (designated as WT-sta and AOE-sta). Subsequently, the algal biomass was harvested via centrifugation, yielding a cell count of approximately 2.6 × 10^8^ cells per sample. The cells were rinsed thrice with sterile water and promptly cryogenically preserved using liquid nitrogen. After passing QC, sequencing was performed using the Illumina platform.

The cleaned data, obtained after processing the raw sequencing reads for enhanced reliability, were aligned to the reference genome of *N. gaditana* B31 using HISAT2 v2.0.5. Based on the location information of gene alignments, the number of reads covering each gene (including newly predicted genes) was counted. The FPKM value for each gene was calculated according to its length to quantify gene expression levels. DESeq2 software (version 1.20.0) was employed for a differential expression analysis between two groups, with a significance threshold set at *p* < 0.05 and |log2(fold change)| greater than 0. Gene Ontology (GO) and Kyoto Encyclopedia of Genes and Genomes (KEGG) enrichment analyses for differentially expressed genes were conducted using the clusterProfiler (version 3.4.4).

Four biological replicates from each algal strain were dispatched to Shanghai Biobay Biologicals Co. for comprehensive liquid chromatography–mass spectrometry (LC-MS) metabolomic profiling.

### 4.8. Chip-Seq and EMSA

The cells of overexpressed strain and WT in logarithmic phase were cross-linked by adding formaldehyde solution (36.5%, Sigma-Aldrich, Darmstadt, Germany) at a final concentration of 1% for 30 min. In the course of the procedure, subject the specimens to sequential cycles of vacuum treatment at pressures ranging from approximately 60 to 80 psi for intervals of 5 min each, followed by the relaxation of the vacuum and thorough homogenization of the cell population. This cycle of vacuum application, relaxation, and cell mixing was executed a total of five times. Subsequently, the cross-linking process was halted through the introduction of a final concentration of 0.125 M glycine, which was allowed to react under vacuum conditions for a duration of 10 min. Following the termination of cross-linking, the algal cells were subjected to two rounds of washing with PBS buffer to eliminate residual cross-linking agents. The protocol of ChIP was followed by Wei and Xu (2018) [43].

High-throughput sequencing was conducted at Beyotime Biotechnology Co., Ltd. (Shanghai, China) utilizing the Novaseq 6000 PE 150 platform. The raw sequencing data underwent stringent filtering to yield clean reads, which were then aligned against the reference genome of *N. gaditana* B31 using the bowtie2 alignment tool. Peaks were identified from the alignment output using MACS2 software (2.2.9.1) with a statistical significance threshold of *p* < 0.01. Following peak identification, comprehensive bioinformatics analyses were performed, including gene annotation, visualization, and functional enrichment analysis of neighboring genes. Additionally, potential motifs within the identified peaks were predicted using the Homer software (v4.8) tool. This systematic approach facilitated the elucidation of genetic regulatory elements associated with the organism under study. An Electrophoretic Mobility Shift Assay (EMSA) was conducted with the manuals of Chemiluminescent EMSA Kit (Beyotime, Shanghai, China, GS009).

## 5. Conclusions

In this study, the NgAP2a overexpression strains of *Nannochloropsis gaditana* were found to accumulate lipids with decreased carbohydrates. However, RNAi strains showed the opposite phenotype. Furthermore, NgAP2a protein was immunoprecipitated and sequenced in overexpression strain. In combination with the transcription and metabolism data, the genes of fatty acid synthesis pathway were screened. And then we found NgAP2a can direct bind to the M1 motif (TCTGCAAAGCYC), which located in the promoter of *KCS* gene (Naga_100083g23). This showed that KCS may be the target of the NgAP2a transcription factor and may then lead to the lipid accumulation of NgAP2a overexpression strains.

## Figures and Tables

**Table 1:** Potential motifs enriched by ChIP-Seq.

Name	Motif	Best Match	*p* Value	% of Targets
M1	TCTGCAAAGCYC	At2g41835(C2H2)/col-At2g41835-DAP-Seq(GSE60143)/Homer(0.692)	1.00 × 10^−13^	1.96
M2	TCGTCAACCC	WRKY62/MA1091.1/Jaspar(0.760)	1 × 10^−13^	13.09
M3	CTTGGGCAAA	PB0133.1_Hic1_2/Jaspar(0.737)	1 × 10^−13^	9.57
M4	CAGCCGAAAC	PDR1/PDR1_YPD/(Harbison)/Yeast	1 × 10^−12^	1.35
M5	ATACCGCAAGTA	RUNX2/MA0511.2/Jaspar	1 × 10^−12^	0.41
M6	GGGAGACTTC	LIN28A(CSD,Znf)/Homo_sapiens-RNCMPT00036-PBM/HughesRNA	1 × 10^−11^	31.88
M7	CCTTCAGCDTGG	Tb_0230(RRM)/Trypanosoma_brucei-RNCMPT00230-PBM/HughesRNA(0.638)	1 × 10^−11^	0.32
M8	AAAAACCTATGG	SRS7(SRS)/colamp-SRS7-DAP-Seq(GSE60143)/Homer(0.685)	1 × 10^−12^	2.37

Note: *p*-value/log *p*-value: the hypergeometric test *p*-value of the input peak relative to the background enrichment to the peak; % of targets: the proportion of the peak that contains the motif; Y: C/T; D: G/A/T. Best Match/Details: De novo is the closest known motif, and it gives detailed data sources.

## Data Availability

The RNA sequencing read data were deposited in the GenBank SRA database under the accession number PRJNA1135349.

## Supplementary Materials

The following supporting information can be downloaded at: https://www.mdpi.com/article/10.3390/ijms251910305/s1.
