# Supplementary figures and images for: NgAP2a Targets KCS Gene to Promote Lipid Accumulation in Nannochloropsis gaditana
Source: Int J Mol Sci. 2024 Sep 25;25(19):10305. doi: 10.3390/ijms251910305 (PMC11477109; doi:10.3390/ijms251910305)

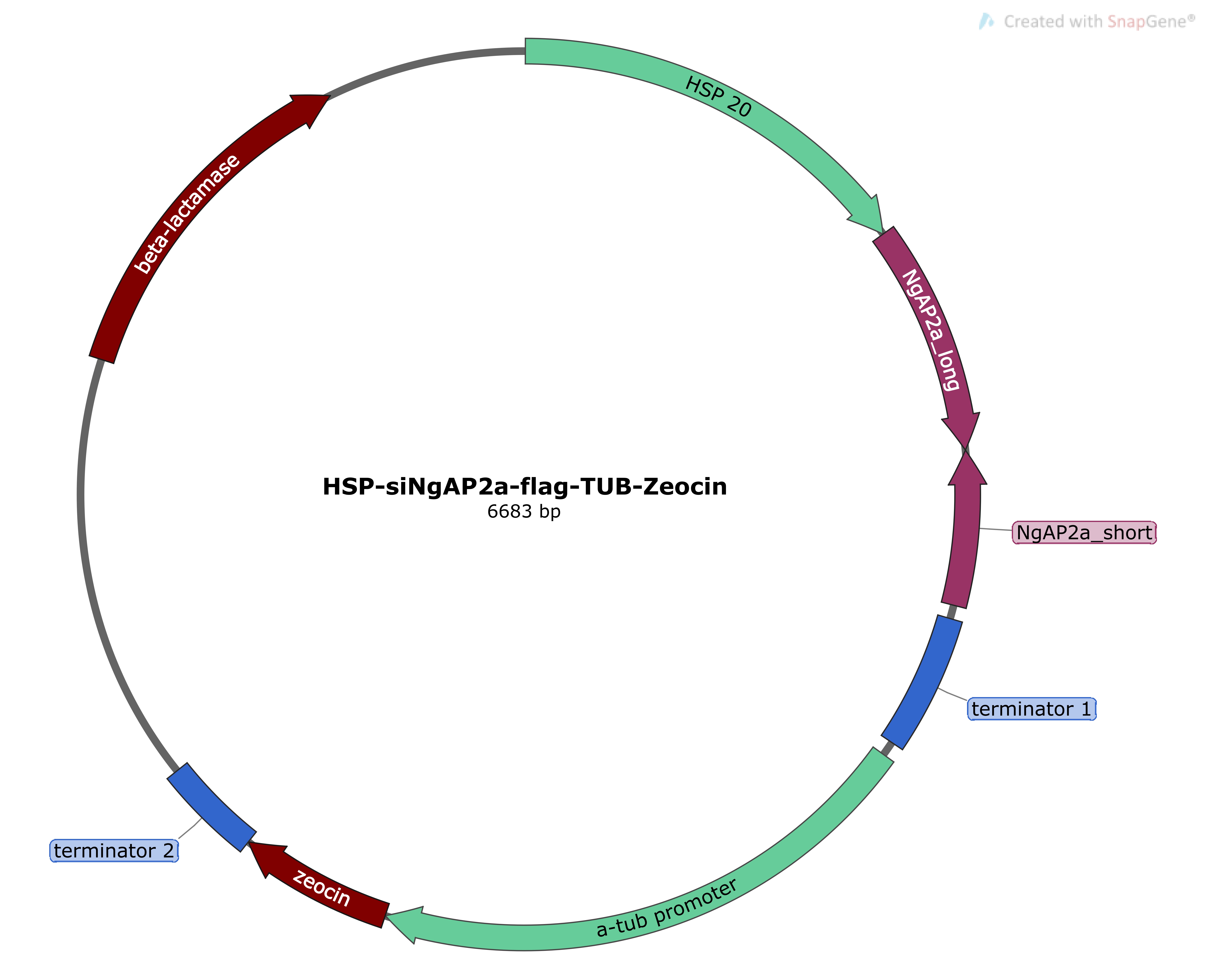

Supplement: Supplementary file 1 [file ijms-25-10305-s001.zip › Figure S1.jpg]

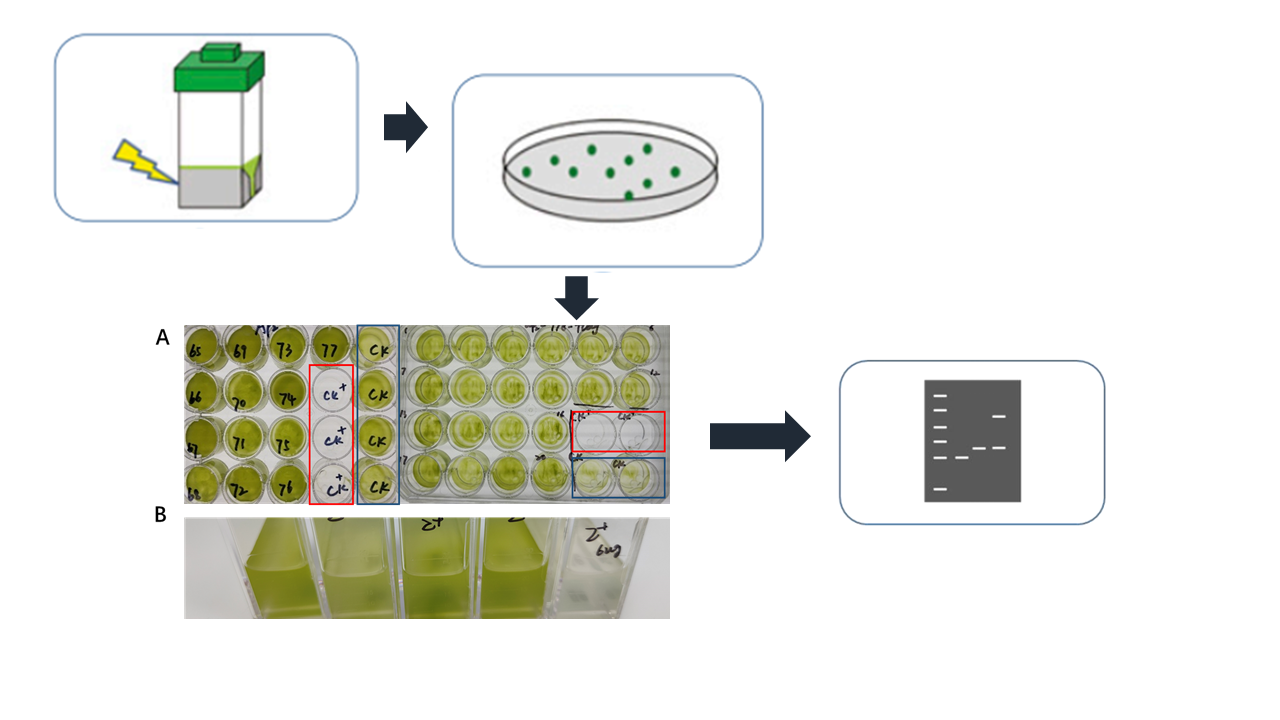

Supplement: Supplementary file 1 [file ijms-25-10305-s001.zip › Figure S2.tif]
